# Supplementary figures and images for: NuMY—A qPCR Assay Simultaneously Targeting Human Autosomal, Y-Chromosomal, and Mitochondrial DNA
Source: Genes (Basel). 2023 Aug 18;14(8):1645. doi: 10.3390/genes14081645 (PMC10454206; doi:10.3390/genes14081645)

**A. mtND1**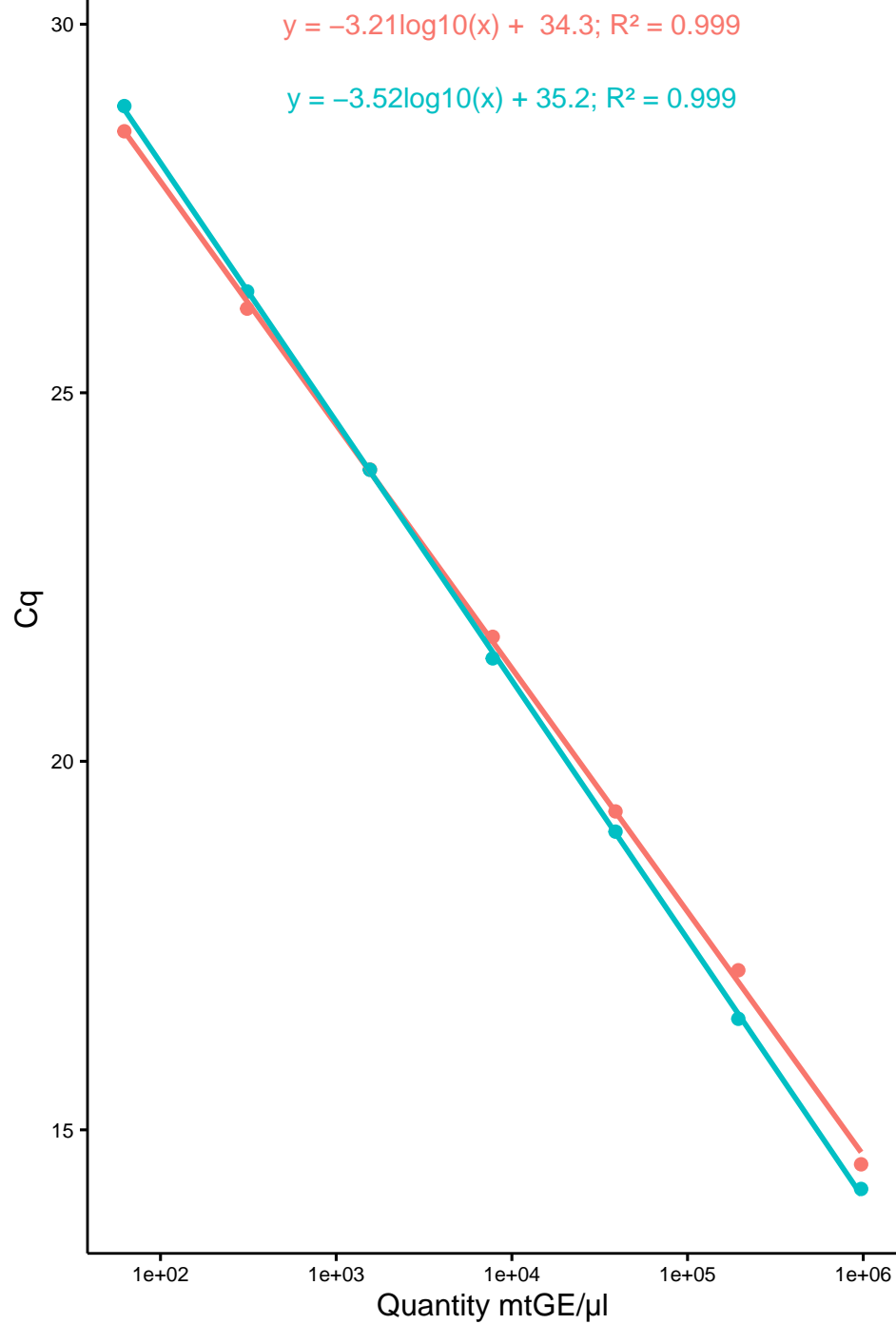**B. YRS**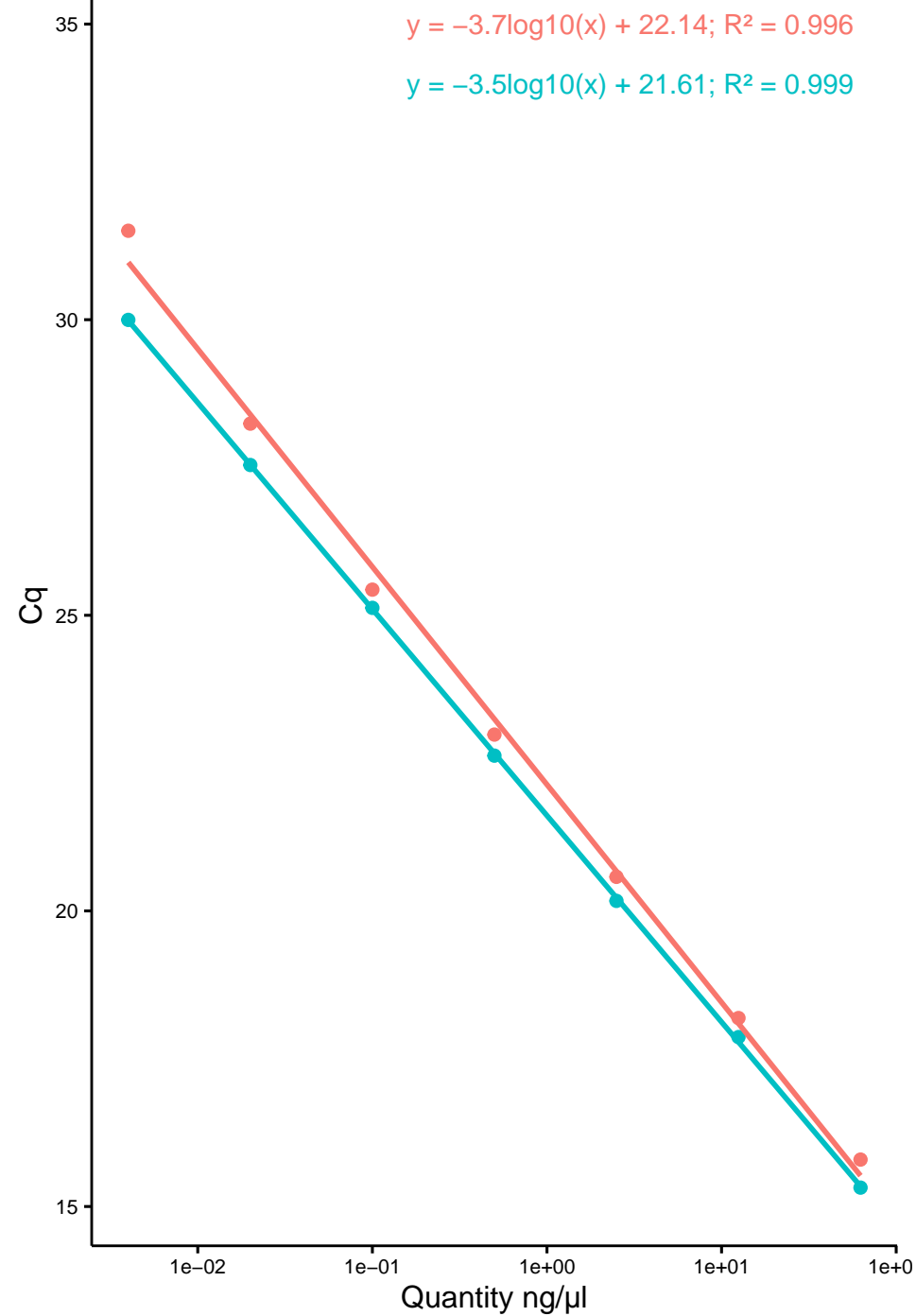**C. nuRNU**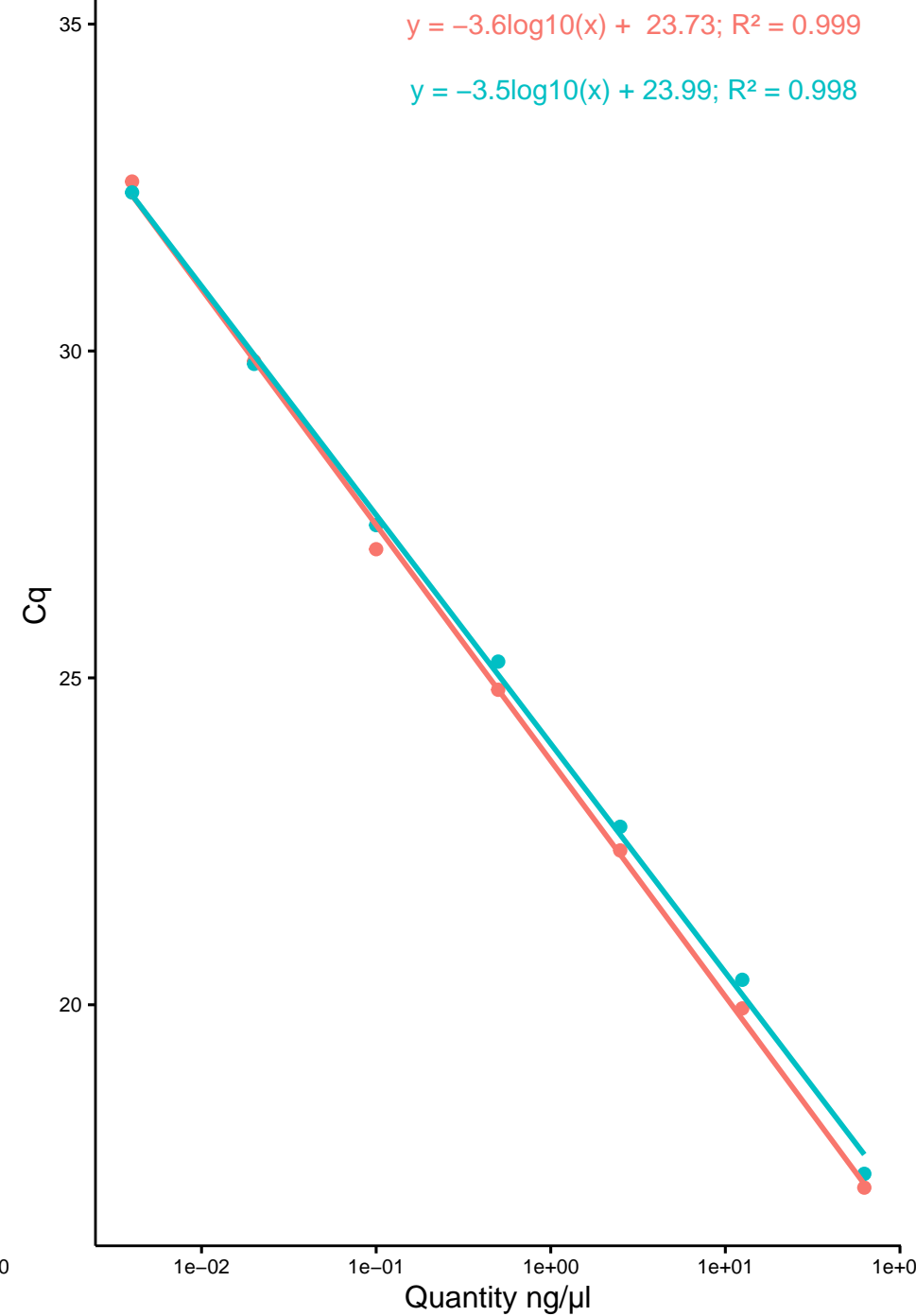

● Multiplex ● Singleplex

Supplement: Supplementary file 1 [file genes-14-01645-s001.zip › SupplementaryFigS1.pdf]
